# Predicting risk of early-onset sepsis in low-resource neonatal units using routine healthcare data: development and evaluation of multivariable statistical and machine learning models

**DOI:** 10.1136/bmjpo-2025-003617

**Published:** 2025-09-26

**Authors:** Ed Lowther, Nushrat Khan, Mario Cortina-Borja, Gwendoline Lilly Chimhini, Samuel R Neal, Marcia Mangiza, Felicity Fitzgerald, Michelle Heys, Simbarashe Chimhuya

**Affiliations:** 1 UCL Advanced Research Computing Centre, University College London, London, UK; 2 Department of Primary Care and Public Health, Imperial College London, London, UK; 3 Population, Policy and Practice Research and Teaching Department, University College London Great Ormond Street Institute of Child Health, London, UK; 4 Neonatal Unit, Sally Mugabe Central Hospital, Harare, Zimbabwe; 5 Department of Child Adolescent and Women's Health, Faculty of Medicine and Health Sciences, University of Zimbabwe, Harare, Zimbabwe; 6 Infectious Diseases, Imperial College London - South Kensington Campus, London, UK; 7 The Health Research Unit Zimbabwe (THRU ZIM), Biomedical Research and Training Institute, Harare, Zimbabwe

**Keywords:** Microbiology, Neonatology

## Abstract

**Background:**

Neonatal sepsis is a major cause of morbidity and mortality in low-resource settings and accurate, context-appropriate diagnostic methods are urgently needed to improve clinical outcomes.

**Methods:**

We used data collected using Neotree, an open source digital health intervention tool, from neonates admitted to Sally Mugabe Central Hospital in Harare between February 2021 and September 2024 to model a composite outcome variable comprised of senior clinician-assigned diagnosis at discharge or cause of death and blood culture test results. Three statistical and machine learning algorithms were developed, tuned where appropriate using cross-validation and evaluated.

**Results:**

In total, 917 cases of early-onset neonatal sepsis were identified among the 18 345 neonates in our study sample, comprising 664 cases of clinician diagnosis and 253 positive blood culture results. With area under the receiver operating characteristic curve as a metric, LightGBM, a machine learning gradient-boosted tree classifier, performed marginally better (0.712; 95% CI 0.673 to 0.75) than logistic regression (0.687; 95% CI 0.646 to 0.728) on a held-out evaluation dataset. A simple and easily interpretable machine learning model, the *k*-neighbours classifier, offered comparable performance (0.699; 95% CI 0.662 to 0.736).

**Conclusions:**

This study explored the potential advantages of using machine learning in the triage of neonates at risk of sepsis in low-resource settings where gold-standard blood culture test results are often unavailable. While the differences in performance metrics were not statistically significant, the machine learning approaches in our study offer other advantages including more intuitive predictions and the ability to handle missing data without imputation.

**WHAT IS ALREADY KNOWN ON THIS TOPIC**

- Neonatal sepsis is a major cause of newborn deaths in low-resource settings, a problem compounded by shortages of trained healthcare staff and laboratory diagnostics. Limited availability of data impedes modelling studies of neonatal sepsis in such settings.

**WHAT THIS STUDY ADDS**

- Using routine healthcare data from ~18 000 newborns collected at admission to a newborn care unit in a tertiary hospital in Zimbabwe, we derive clinical diagnostic algorithms for early neonatal sepsis using three contrasting statistical and machine learning models, exploring trade-offs between accuracy and interpretability.

**HOW THIS STUDY MIGHT AFFECT RESEARCH, PRACTICE OR POLICY**

- We show how data-driven clinical support tools may be able to offer early predictions of neonatal sepsis in this context. While these are yet to be integrated in a digital health system, we assess ways of doing so that may help clinicians understand these predictions intuitively. We also suggest data quality improvements that may boost model performance in future research.

## Background

 Neonatal sepsis leads to ~800 000 deaths annually, mostly in low-resource settings (LRS).^1 2^ Where laboratory diagnostics are scarce, as in many sub-Saharan African settings, guidelines based on clinical features alone are key to supporting clinicians to identify newborns either with or at risk of sepsis.^3 4^ Most current clinical guidelines involve a simple scoring system where each feature is assigned equal weight,^5 6^ although some features carry much higher risk than others.^7^ Allocating equal importance to all risk factors often leads to overtreatment, with concomitant generation of antimicrobial resistance and unnecessary resource use.^3 4^ Developing a model with weighting for higher risk signs and allowing more complexity than simple score summation could improve clinical management for millions of neonates worldwide.

In high-resource settings, online sepsis risk calculators have been used to safely reduce neonatal antibiotic treatment.^8^ However, the incidence of bloodstream infection in these tools’ derivation cohorts is ~40 times lower than in LRS, likely precluding their applicability^9^. Where sufficiently large quantities of quality routine healthcare data are available, artificial intelligence techniques are being used to develop improved prediction and diagnostic models for a range of clinical conditions, allowing for personalised risk evaluation.^10^,^12^ To date, data of this granularity have rarely been available from patient cohorts in LRS. There is potential, however for substantial improvements in risk prediction and clinical care, particularly around antibiotic prescribing, if high quality data were available.^13^

Neotree (henceforth, ‘the app’) is a low-cost, open source digital health intervention system that supports data collection, education of healthcare professionals and newborn care at the hospital bedside, which is being implemented in two tertiary level hospitals in Zimbabwe and one hospital in Malawi. Data are entered via an interface running on Android tablets into the app by senior resident medical officers (SRMOs), second year doctors rotating through neonates with 3–4 month placements. The app is designed to support them in identifying signs and standardising predictor assessments, for example, with images. The Neotree implementation and development protocol and description of community engagement and app co-development can be found elsewhere.^14 15^ Its development and pilot implementation^14 16 17^ paved the way for previous work, in which a Delphi process was used to identify variables for inclusion in LRS neonatal sepsis prediction models,^18^ and a traditional logistic regression model was developed to predict the risk of sepsis.^19^ However, this model had poor specificity at acceptable sensitivity, meaning it was unlikely to reduce unnecessary antibiotic use. A scoping review of clinical prediction models for neonatal sepsis in LRS found few models have been developed in sub-Saharan Africa.^20^

In this study, we aimed to investigate how machine learning techniques could inform the development and evaluation of a clinical prediction model to improve diagnosing neonatal sepsis using data from a large tertiary neonatal unit in Zimbabwe collected using the app.

## Methods

### Setting

Sally Mugabe Central Hospital (SMCH) in Harare is a large, central, public teaching hospital with approximately 15 000 deliveries a year. The 100-bed, tertiary level neonatal unit is the nationwide surgical centre and accepts preterm neonates from the northern half of the country. The neonatal unit follows national guidelines for identifying neonates with clinical features of or risk factors for sepsis, taking blood cultures and commencing antibiotics (EDLIZ 2020, online supplemental table 1). Senior clinicians (senior paediatric registrars or neonatal consultants) carry out daily ward rounds subsequent to Neotree-supported admission by SRMOs and decide on final diagnosis captured at discharge within Neotree on the basis of clinical progress. Blood culture tests are performed at the onsite laboratory using an automated BD BACTEC machine, with results captured in the app.

### Data preparation and outcome definition

Our dataset included 19 233 total admission and discharge records, of which 3645 included at least one valid blood culture result. As our focus was on early-onset neonatal sepsis, we excluded cases where either age had not been recorded (n=387) or age at admission was more than 72 hours (n=501), leaving 18 345 cases. Among these admissions, 27.6% (n=5063) had low birth weight (ranging 1.5–2.5 kg), 7.3% (n=1348) had very low birth weight (ranging 1–1.5 kg) and 3% (n=562) had extremely low birth weight (less than 1 kg) according to WHO categories.

**Figure 1:**
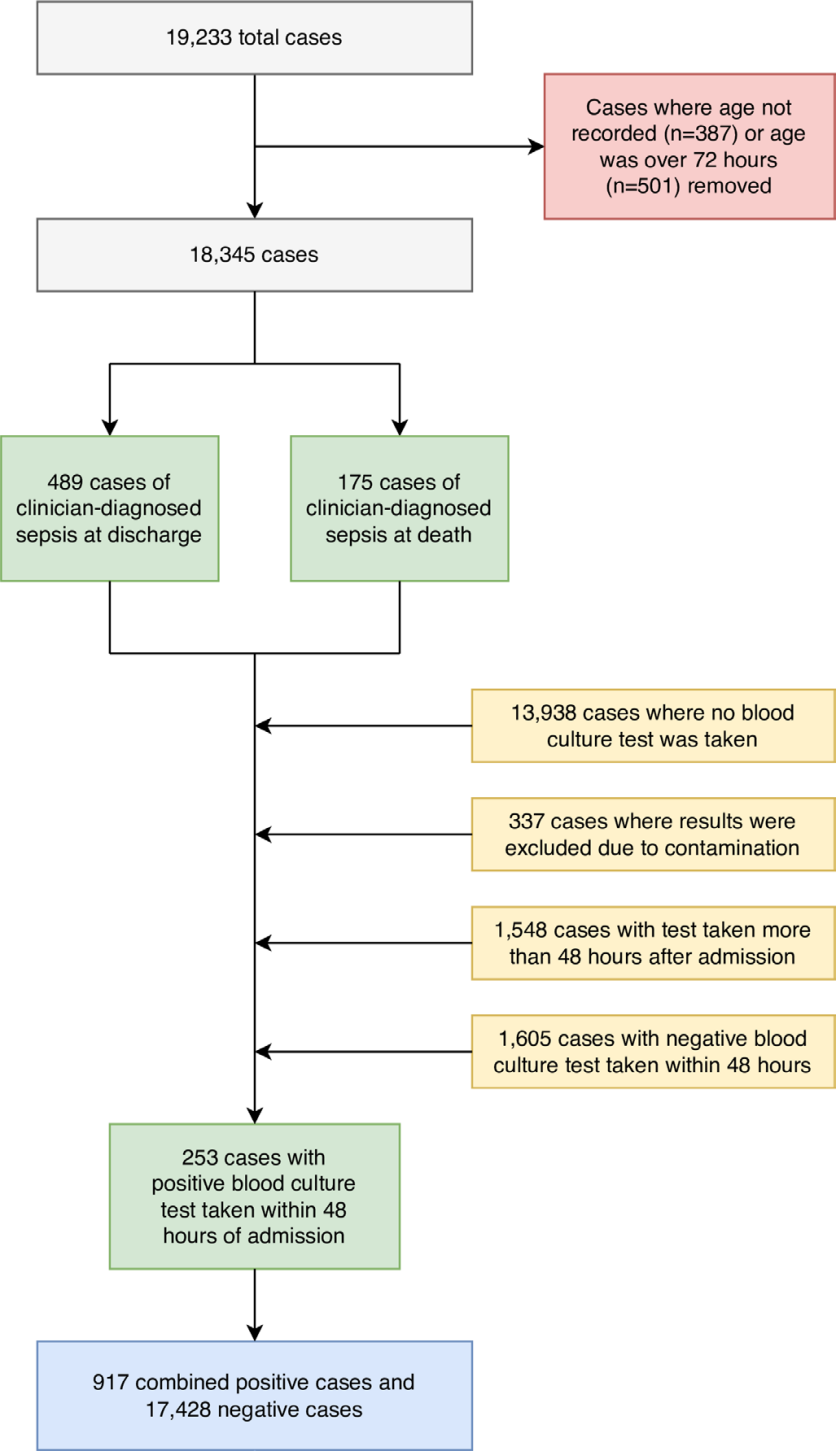
Flow chart of data preparation and construction of composite outcome variable.

We defined early-onset neonatal sepsis as meeting one of the following conditions: (a) senior clinician diagnosis of sepsis on discharge (n=489) or cause of death listed by clinicians as sepsis (n=175); (b) a valid positive blood culture test result, with the sample having been taken no more than 48 hours after admission (n=253). Our outcome variable therefore comprised 917 positive cases and 17 428 negative cases (figure 1). Blood culture test results were excluded from the analysis on detection of either coagulase negative staphylococcus or well-recognised contaminants such as *Corynebacterium, Bacillus* sp and *Micrococcus*. The diagnosis of sepsis at discharge will have been made by either a registrar with at least 5 years’ paediatric experience or a consultant. There may be a degree of variation in individual decision-making at a senior level, but as the senior doctors cover a fortnight each in turn on the rota, this is likely to even out over time.

The dataset comprises all records entered into the app at SMCH starting February 2021, when a shift in operating procedures resulted in blood culture tests becoming more widely available. Total sample size exceeds the criteria described in the study by Riley *et al*.^21^

### Predictors

Our approach was informed by previous work to explore the predictive value of clinical and demographic data using regression analyses.^19^ 24 risk factors were considered for inclusion: Apgar scores at 1 min, 5 min and 10 min, age at admission, gender, gestational age, birth weight, temperature at admission, heart rate, oxygen saturation in air, blood sugar, type of birth (eg, single, twin, triplet), duration of rupture of membranes, skin condition, presence of danger signs,^22^ respiratory distress, work of breathing, alertness, umbilicus condition, newborn’s appearance/colour, respiratory rate, vomiting, abdominal examination and fontanelle.

All but three of the continuous variables had some missing values. Two variables had very high levels of missingness: blood sugar and Apgar score at 10 min, at 98.7% and 90.1%, respectively, and were therefore excluded from our analysis, resulting in a final set of 22 risk factors. Missingness in the remaining continuous variables ranged from 1227 cases for the Apgar score at 5 min (6.9%) to 54 cases for birth weight (0.3%). Rows with missing age values were removed in data preprocessing, and the only continuous variables with no missing values were gestational age and temperature.

The number of missing values for categorical variables ranged from 18 190 for skin condition (99.2%) to 22 cases for umbilicus (0.12%). In at least some cases, missingness is likely to be of clinical relevance, for example, signifying that the neonate was breathing normally rather than enduring breathing difficulties that went unrecorded. To avoid discarding potentially relevant information of this kind, for all categorical variables, missingness was encoded as a value.

Some data fields in the app are designed to accept multiple inputs, in cases where multiple symptoms may be present at the time of examination. For example, for ‘signs of respiratory distress’ clinicians may enter a combination of one or more of seven different values: ‘nasal flare’, ‘grunting’, ‘chest indrawing’, ‘head nodding’, ‘tracheal tug’, ‘stridor’ and ‘gasping’. The distribution of these selections has a long tail; many cases have been assigned unusual combinations, including 43 that are unique. To reduce model complexity, we therefore grouped and simplified the values of these multiple choice fields; the signs of respiratory distress variable, for example, were reduced to presence or absence of one or more signs or whether no value had been recorded. Five other variables were simplified in a similar way: presence of danger signs, umbilicus condition, abdominal examination, skin condition and vomiting.

All categorical variables were converted to integers, so that both continuous and categorical variables could be scaled to have a mean value of zero and SD of one and also to capture information about ordinal categorical variables that might otherwise be missed by the models, for example, that the values for work of breathing range in order of severity from mild to moderate to severe.

### Model development

The baseline demographic and clinical characteristics of neonates in our dataset are described in table 1. The dataset was randomly split into a training and evaluation set, with ~25% in the evaluation set. Stratification was used to ensure that the proportion of positive and negative cases in each set was comparable. All model development took place using the training dataset only, with the evaluation set held back for subsequent model evaluation.

**Table 1:** Baseline demographic and clinical characteristics of the dataset

Predictor	Levels	All cases (n=18 345)	Composite outcome positive (n=917)	Composite outcome negative (n=17 428)
Age (hour); median(Q1–Q3)	–	2.0 (1.0–7.0)	3.0 (1.0–18.0)	2.0 (1.0–6.0)
Gender; n (%)	Female	8222 (44.82)	399 (43.51)	7823 (44.89)
	Male	10 089 (55.00)	516 (56.27)	9573 (54.93)
	Ambiguous	34 (0.19)	2 (0.22)	32 (0.18)
Gestational age; median(Q1–Q3)	–	38 (35-40)	38 (34-39)	38 (35-40)
Birth weight; median(Q1–Q3)	–	2710 (2000–3200)	2700 (1850–3200)	2725 (2000–3200)
	Missing	54 (0.29)	2 (3.70)	52 (96.30)
Temperature at admission; median(Q1–Q3)	–	36.8 (36.6–37.0)	36.8 (36.6–37.0)	36.8 (36.6–37.0)
Heart rate; median(Q1–Q3)	–	138.0 (129.0–147.0)	140.0 (129.0–150.0)	138.0 (129.0–146.0)
	Missing	93 (0.51)	3 (3.23)	90 (96.77)
Apgar score at 1 min; median(Q1–Q3)	–	7 (5–8)	7 (5–8)	7 (5–8)
	Missing	1183 (6.45)	86 (7.27)	1097 (92.73)
Apgar score at 5 min; median(Q1–Q3)	–	8 (7–9)	8 (7–9)	8 (7–9)
	Missing	1227 (6.69)	89 (7.25)	1138 (92.75)
Respiratory rate; median(Q1–Q3)	–	38 (29–58)	40 (30–62)	38 (28–58)
	Missing	140 (0.76)	4 (2.86)	136 (97.14)
Oxygen saturation in air; median(Q1–Q3)	–	92.0 (87.0–96.0)	90.0 (86.0–95.0)	92.0 (87.0–96.0)
	Missing	341 (1.86)	17 (4.99)	324 (95.01)
Type of birth; n (%)	Singleton	16 381 (89.29)	840 (91.60)	15 541 (89.17)
	Twin	1895 (10.33)	72 (7.85)	1823 (10.46)
	Triplet	69 (0.38)	5 (0.55)	64 (0.37)
Premature rupture of membrane; n (%)	Yes	1264 (6.89)	151 (16.47)	1113 (6.39)
	No rupture	15 743 (85.82)	715 (77.97)	15 028 (86.23)
	Missing	1338 (7.29)	51 (5.56)	1287 (7.38)
Skin condition; n (%)	Condition present	155 (0.84)	8 (0.87)	147 (0.84)
	Missing	18 190 (99.16)	909 (99.13)	17 281 (99.16)
Work of breathing; n (%)	Mild	3543 (19.31)	203 (22.14)	3340 (19.16)
	Moderate	4011 (21.86)	253 (27.59)	3758 (21.56)
	Severe	931 (5.07)	60 (6.54)	871 (5.00)
	Missing	9860 (53.75)	401 (43.73)	9459 (54.27)
Signs of respiratory distress; n (%)	Signs present	9042 (49.29)	537 (58.56)	8505 (48.80)
	Missing	9303 (50.71)	380 (41.44)	8923 (51.20)
Danger signs*; n (%)	Signs present	5252 (28.63)	332 (36.21)	4920 (28.23)
	Missing	13 093 (71.37)	585 (63.79)	12 508 (71.77)
Alertness; n (%)	Alert	14 907 (81.26)	694 (75.68)	14 213 (81.55)
	Coma	167 (0.91)	2 (0.22)	165 (0.95)
	Convulsions	66 (0.36)	8 (0.87)	58 (0.33)
	Lethargic	2979 (16.24)	193 (21.05)	2786 (15.99)
	Irritable	226 (1.23)	20 (2.18)	206 (1.18)
Colour; n (%)	Pink	17 674 (96.34)	877 (95.64)	16 797 (96.38)
	Blue	481 (2.62)	29 (3.16)	452 (2.59)
	Pink/white	1 (0.01)	0 (0.00)	1 (0.01)
	White	133 (0.72)	4 (0.44)	129 (0.74)
	Yellow	31 (0.17)	6 (0.65)	25 (0.14)
	Yellow/white	1 (0.01)	1 (0.11)	0 (0.00)
	Missing	24 (0.13)	0 (0.00)	24 (0.14)
Umbilicus; n (%)	Normal	17 619 (96.04)	877 (95.64)	16 742 (96.06)
	Abnormal	726 (3.96)	40 (4.36)	686 (3.94)
	Missing	22 (0.12)	0 (0.00)	22 (0.13)
Vomiting; n (%)	Small milky possets after feeds (normal)	79 (0.43)	6 (0.65)	73 (0.42)
	Yes (all feeds/ blood/ green)	249 (1.36)	23 (2.51)	226 (1.30)
	No	18 017 (98.21)	888 (96.84)	17 129 (98.28)
	Missing	27 (0.15)	0 (0.00)	27 (0.15)
Abdomen check; n (%)	Normal	17 713 (96.55)	878 (95.75)	16 835 (96.60)
	Abnormal	632 (3.45)	39 (4.25)	593 (3.40)
	Missing	49 (0.27)	2 (0.22)	47 (0.27)
Fontanelle; n (%)	Flat	18 187 (99.14)	908 (99.02)	17 279 (99.15)
	Bulging	92 (0.50)	3 (0.33)	89 (0.51)
	Sunken	66 (0.36)	6 (0.65)	60 (0.34)

* Danger signs as per WHO Emergency Triage Assessment and Treatment guidelines relevant for newborns, including grunting, cyanosis, convulsions, capillary refill time <3 s, trunk feels cold, weak femoral pulses.

We developed three models for comparison: logistic regression, a *k*-neighbours classifier and a gradient-boosted trees classifier. The *k*-neighbours algorithm is a simple machine learning method, available in Python via the scikit-learn library,^23^ that defines the probability of a test case belonging to a class as the proportion of the *k* most similar cases from the training data that were in that class. Gradient-boosted tree algorithms involve building a sequence of decision trees, with each aiming to correct the errors of its predecessors; we applied Microsoft Research’s LightGBM, which was designed to deliver computational efficiency^24^ and has demonstrated promise in various diagnostic settings.^25^,^27^ The performance of LightGBM may be improved by carefully choosing hyperparameter values, for example “reg_alpha” which reduces the impact of less-informative predictors. We defined a search space for a selection of LightGBM hyperparameters and optimised their value using cross-validation on the training dataset with five folds and the Tree-structured Parzen Estimator algorithm, available via the Optuna Python library.^28^ We selected the mean area under the receiver-operating characteristic curve (AUROC) for the five cross-validation folds as the metric to optimise, stratifying the folds by outcome variable. The *k*-neighbours classifier has just one hyperparameter, *k*; we used the same approach to optimise its value.

Neither logistic regression nor the *k*-neighbours classifier can be fit on data that includes missing values in continuous predictor variables without either first imputing those values or sacrificing granularity by grouping them into buckets (where one bucket could represent missingness). Conversely, LightGBM handles missing values by treating them like any other continuous value, as it seeks to minimise training error. To enable a fair comparison of the three models, we initially developed and evaluated the models on the dataset with rows including missing continuous values removed (n=1750). To avoid the risk that the distance calculation inherent in *k*-neighbours classification attends to variables that may take higher values, all variables in the training dataset were scaled to have a mean of zero and a SD of one. We subsequently re-ran the analysis with LightGBM only using the data without scaling and with missing continuous values reinstated. The counts of participants and outcomes in each scenario are reported in online supplemental table 2.

### Model evaluation

The evaluation metric used for model predictions on the held-out evaluation dataset was AUROC, which has been shown to perform effectively even when the outcome variable is imbalanced.^29^ In the initial model comparison step that involved scaling the data, the mean and SD of the training dataset were used to scale both the training and evaluation datasets, employing the Mann-Whitney U test to identify any differences in the distribution of predictors between the two datasets. We obtained the predicted probabilities for the scaled evaluation dataset using each model’s implementation of the ‘predict_proba’ method and used the DeLong statistical test both to derive CIs for their associated AUROC values and assess the significance of differences in model performance,^30^ setting the significance level to be 5%. Analysis of the importance of the predictor variables to each model’s predictions was undertaken using Shapley values, a concept with roots in cooperative game theory, which may be used to quantify the utility of each predictor variable used by any statistical or machine-learning model.^31^

### Software

We used Python V.3.9.19 with pandas V.2.2.2 for data preprocessing, scikit-learn library^23^ V.1.5.1 for logistic regression and *k*-neighbours classification, LightGBM^24^ V.4.5.0, V.3.6.1 of the hyperparameter search library Optuna^28^ and SHAP^31^ V.0.46.0 for calculation of Shapley values, among other open-source Python tools. DeLong statistical tests were performed using the pROC library^32^ V.1.18.5 with R^33^ V.4.4.1. For a full list of dependencies, please see the GitHub repository associated with this project (https://github.com/edlowther/neotree-analysis).

### Patient and public involvement

While patients and the public were not specifically involved in the work described here, Neotree has a strong community engagement component in our work in Zimbabwe, including mothers’ groups who support the codesign of the app, to ensure we best serve the needs of families at our sites. They are involved in both the ongoing codesign and dissemination of our findings.^14 15^

## Results

**Figure 2:**
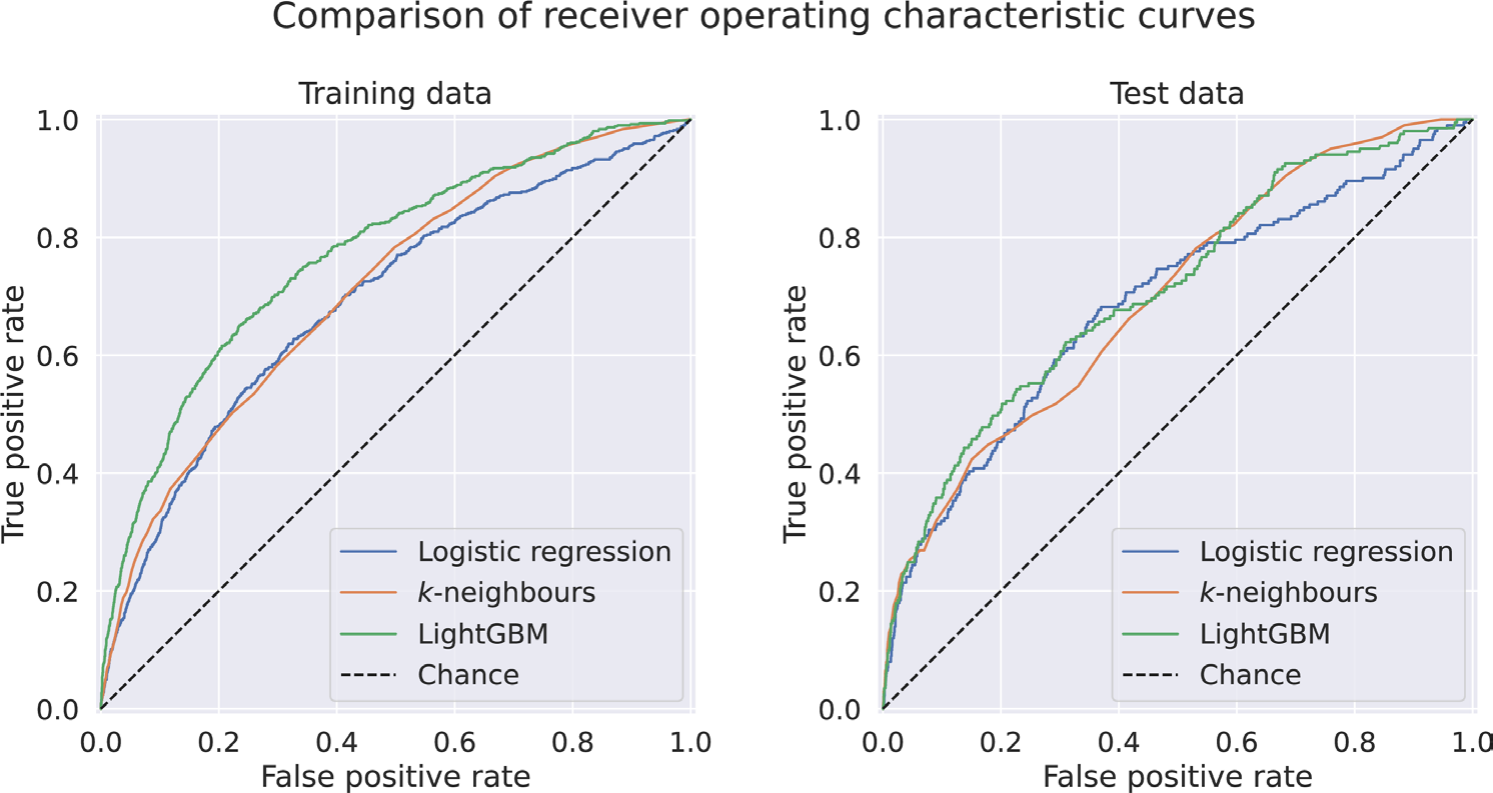
Receiver operating characteristic curves for logistic regression, k-neighbours classifier and LightGBM models for development and evaluation datasets.

None of the pairwise comparisons of ROC curves on the evaluation data between the three models (figure 2) using the DeLong statistical test met our threshold for statistical significance of 5% (logistic regression, *k*-neighbours classification: p=0.42; *k*-neighbours classification, LightGBM: p=0.34; logistic regression, LightGBM: p=0.09). Model optimisation resulted in a value of *k*=459 neighbours for the *k*-neighbours classifier; full details of the search space for all hyperparameters and the results of tuning the LightGBM model are available on the project’s GitHub repository.

**Figure 3:**
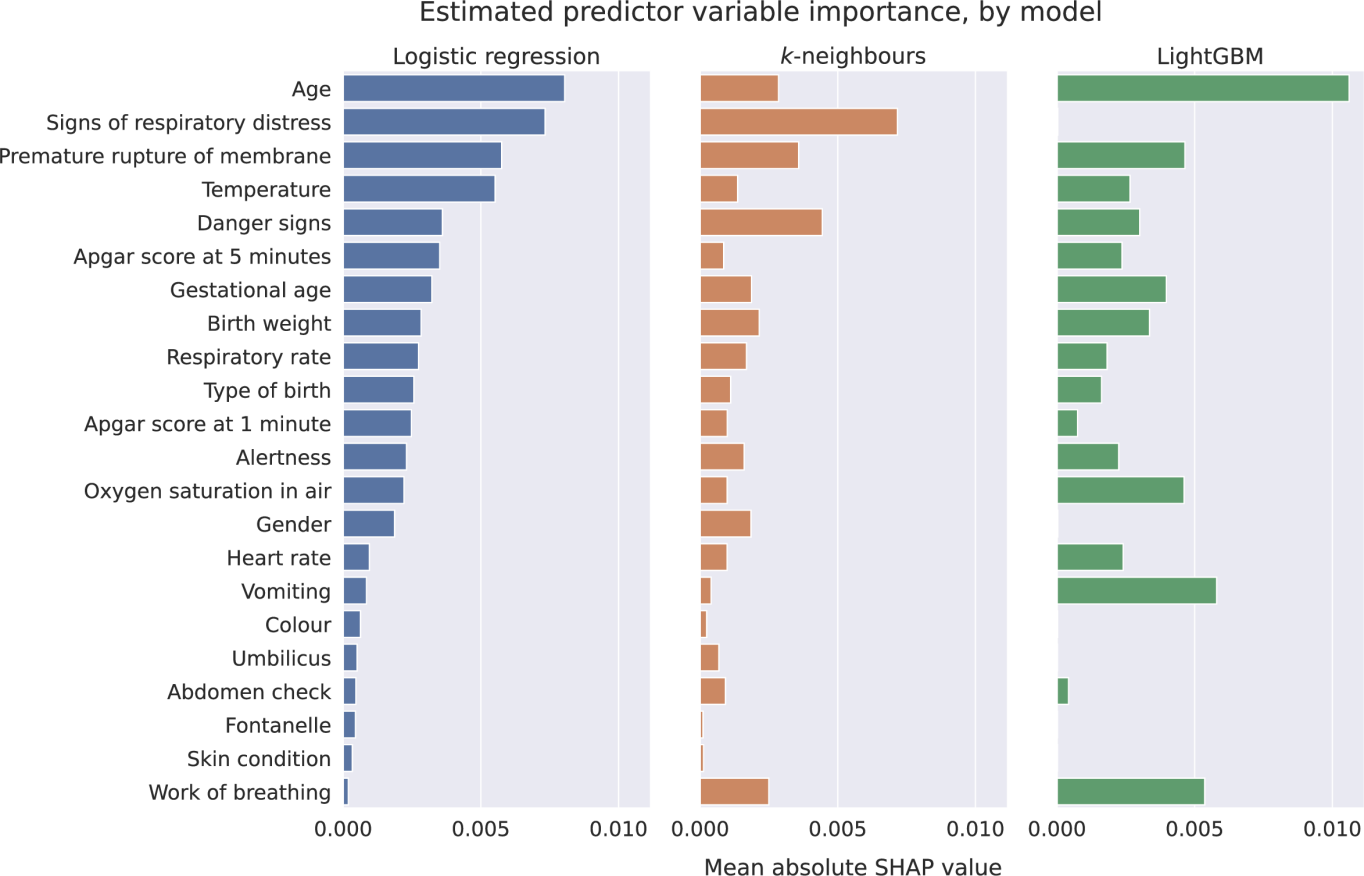
Analysis of estimated mean absolute Shapley (SHAP) values as a proxy for predictor variable importance reveals some contrasts between the three modelling approaches, for example, that respiratory distress is the most impactful variable for k-neighbours classification yet has no influence on LightGBM predictions. Variables with disproportionate levels of homogeneity (skin condition, which is missing in 99.2% of cases and umbilicus, which is normal in 96.0%) have little influence on any of the models. The work of breathing variable was of minimal importance for the logistic regression model but above average importance for the other two classifiers.

Computation of mean absolute Shapley values for each model revealed that the most important predictor variable for both the logistic regression and LightGBM models was age at admission, whereas for the *k*-neighbours classifier, it was the signs of respiratory distress variable which, in contrast, the LightGBM model found to be much less important (figure 3). Other differences in emphasis include work of breathing and vomiting, which feature in LightGBM’s top three but are much lower in importance for logistic regression. Premature rupture of membranes appears in the top four for all of the models. Mean absolute Shapley values tended towards zero for some variables for each of the three models; however, this was observed more frequently with the LightGBM model than with the other two (mean absolute Shapley values of less than 0.0001 for six predictors compared with none for the other two classifiers). In the initial modelling stage, with missing continuous values removed, Mann-Whitney U test results indicated no significant differences in distributions between the normalised development and evaluation datasets at the 5% level.

Although not directly comparable, LightGBM performance on the dataset with missing continuous values included and without scaling either the continuous or categorical variables was similar in magnitude (AUROC 0.717; 95% CI 0.681 to 0.752) to the above results. There were no statistically significant Mann-Whitney U test results between the training and evaluation datasets in this case. The trade-off between sensitivity and specificity at various thresholds for this model is presented in table 2. If this model were calibrated to target the minimum viable sensitivity of 85% for a hospital-based test suggested in a recent draft target product profile for neonatal sepsis tests by the WHO,^34^ on our evaluation dataset it would have attained 38.9% specificity, falling short of the WHO’s accompanying minimum target for specificity of 80%.

**Table 2:** Evaluation dataset performance metrics at selected decision-making thresholds for LightGBM model trained on data with missing continuous values included and without scaling input data

Threshold	Sensitivity	Specificity	PPV	NPV	+ve likelihood ratio	-ve likelihood ratio
Youden’s Index	0.729	0.606	0.088	0.977	1.852	0.447
WHO minimum sensitivity	0.852	0.389	0.068	0.98	1.393	0.382
WHO preferred sensitivity	0.952	0.186	0.058	0.985	1.169	0.258

NPV, negative predictive value; PPV, positive predictive value.

## Discussion

This analysis marks an important step along the road to developing a context-specific clinical prediction model for neonatal sepsis in LRS using Neotree, an exemplar learning healthcare system,^14^ with the ultimate goal of helping to identify, at the point of admission, the most vulnerable neonates who could benefit from early antibiotic treatment and those who can safely avoid exposure to antibiotic treatment.

Of the models investigated in this study, LightGBM achieved competitive predictive performance with minimal data preprocessing and no need to impute missing continuous values, which may be a great asset in LRS where clinicians are overstretched and may not have time to capture every variable of interest. Implementation of a *k*-neighbours classifier could offer alternative benefits in a clinical setting by displaying intuitive explanations of model predictions, for example, that a patient is at high risk of sepsis because among the *k* most recent similar admissions x% went on to receive a clinician diagnosis of sepsis, and y% underwent a blood culture test, of which z% tested positive. Additionally, by surfacing summary statistics such as the average distance between a new case and its *k* closest predecessors, an interface for a model of this kind could flag the degree to which each new case was unusual, providing the clinician with valuable information that might inform both clinical care and model output interpretation, for example, that model predictions for very unusual cases should be treated with caution.

There are limitations to this study. At present, predictor variables are collected only at one time point (admission), meaning it is not possible to account for changes over time when, for example, a neonate’s condition deteriorates rapidly after admission. This study is limited by a challenge common to many LRSs, limited availability of blood cultures.^35 36^ Blood cultures are a challenging gold standard for neonates even in high resource settings,^37^ so we made a pragmatic decision to include senior clinician diagnosis of early-onset sepsis recorded at discharge within our outcome. Further, data are from a single, urban site. SMCH serves among the most deprived of the area’s population, but the model may not be generalisable to rural areas, and the most vulnerable mothers may not even reach hospital to deliver. Detailed sociodemographic data were not available as part of this analysis, and although our assumption is that these factors are unlikely to have an influence on predictor assessment or outcome assignment, we have not been able to test this assumption. When more than one sibling in a twin/triplet birth is admitted, a multilevel modelling approach that accounts for relevant shared characteristics between siblings may help to improve performance; however, this is not possible under the current data schema where twins/triplets are not linked by a common ID and must therefore be modelled as independent cases. This issue will be considered as we look to improve data quality for future research. The modelling is yet to undergo external validation; this will be the focus of future work using Neotree data from primary healthcare clinics and Kamuzu Central Hospital, Malawi.

More frequent data collection and continued improvements in data validation at the point of entry are likely to benefit forthcoming iterations of this modelling. If this results in a model that surpasses the WHO minimum standards for utility in a hospital setting,^34^ the app could present model outputs to clinicians at the point of data entry, thereby helping to prevent unnecessary treatment. However, safety must remain of paramount importance; the consequence of missing a diagnosis of neonatal sepsis could be death.

An important next step will be stakeholder consultation involving families, clinicians and policy makers about how to define where the thresholds for clinical decision-making of this kind should be set. The cost for families when a neonate is admitted may be severe, including out-of-pocket expenses such as travel, accommodation and separation from a parent (usually the mother) from the rest of the family for the duration of the admission.^38^

## Conclusion

We have used routine data from a low-resource neonatal unit in Zimbabwe to develop a combination of statistical and machine learning algorithms aiming to predict neonatal sepsis from data collected at the point of admission. While the models’ performance does not yet meet the WHO threshold for clinical utility, we have demonstrated the potential for digital tools such as Neotree to support the development and delivery of contextually appropriate clinical prediction algorithms despite the unit’s low resources. Improving diagnostic accuracy for neonatal sepsis in LRSs is crucial for mitigating against further generation of antimicrobial resistance and concomitant mortality.

## Supplementary material

10.1136/bmjpo-2025-003617 online supplemental file 1

## Data Availability

Data are available upon reasonable request. While an open source anonymised research database is planned as part of the wider Neotree project, currently, sharing of deidentified individual participant data will be considered on a case-by-case basis in line with Zimbabwean data protection laws
