# Supplementary material for: Predicting risk of early-onset sepsis in low-resource neonatal units using routine healthcare data: development and evaluation of multivariable statistical and machine learning models
Source: BMJ Paediatr Open. 2025 Sep 26;9(1):e003617. doi: 10.1136/bmjpo-2025-003617 (PMC12481410; doi:10.1136/bmjpo-2025-003617)
Supplement: online supplemental file 1 [file bmjpo-9-1-s001.docx]

**Supplementary Table 1:** **Essential Drugs List in Zimbabwe 2020 (EDLIZ 2020) Neonatal sepsis guidelines: criteria to be used for commencing antibiotics and taking blood cultures (if available)[1]**

| Major Criteria  (Start antibiotics if any of these present) | Minor Criteria  (Start antibiotics if any two available) |
| --- | --- |
| 1. Confirmed sepsis or chorioamnionitis in mother  2. Confirmed or suspected sepsis in twin  3. Seizures  4. Severe Respiratory Distress in a term infant  5. Signs of shock | **Antenatal:**  1. Rupture of membranes >18h  2. Spontaneous Preterm Birth  3. Group B Streptococcus (GBS) sepsis in previous baby or documented GBS carriage in this pregnancy (urine or vaginal swab) |
|  | **Natal**  4. Born Before Arrival  5. Meconium stained Liquor |
|  | **Postnatal**  6. Respiratory distress that is not obviously related to:   - environmental hypothermia - “delayed transition to extra-uterine life” i.e. mild to moderate respiratory distress apparent soon after birth that is improving with time.   7. Hypoxia  8. Apnoea  9. Hypoglycaemia/Hyperglycaemia not otherwise explained  10. Temperature instability not explained by environmental factors  11. Acidosis not obviously related to HIE  12. Unexplained bleeding or thrombocytopenia  13. Mild encephalopathy/Altered responsiveness  14. Altered tone not otherwise explained  15. Feed intolerance/feeding difficulty  16. Abnormal heart rate (<90 or >160)  17. Jaundice in first 24 hours |

[1] Ministry of Health and Child Care Z. Essential Medicines List and Standard Treatment Guidelines for Zimbabwe., **2020**.

Supplementary Table 2: Number of participants and outcome events in each dataset

|  |  | Individuals (% of full dataset) | Outcomes (% of training/test dataset) |
| --- | --- | --- | --- |
| Model comparison, missing continuous values removed | Training dataset | 12,446 (75%) | 604 (4.85%) |
|  | Evaluation dataset | 4,149 (25%) | 201 (4.84%) |
| Final analysis, LightGBM only | Training dataset | 13,758 (75%) | 688 (5.00%) |
|  | Evaluation dataset | 4,587 (25%) | 229 (4.99%) |
