# Supplementary material for: Predicting risk of early-onset sepsis in low-resource neonatal units using routine healthcare data: development and evaluation of multivariable statistical and machine learning models
Source: BMJ Paediatr Open. 2025 Sep 26;9(1):e003617. doi: 10.1136/bmjpo-2025-003617 (PMC12481410; doi:10.1136/bmjpo-2025-003617)
Supplement: Supplementary file 2 [file bmjpo-2025-003617.reviewer_comments.pdf]

PEER REVIEW HISTORY

BMJ Paediatrics Open publishes all reviews undertaken for accepted manuscripts. Reviewers are asked to complete a checklist review form and are provided with free text boxes to elaborate on their assessment. These free text comments are reproduced below.

ARTICLE DETAILS

**TITLE (PROVISIONAL)**	Predicting risk of early-onset sepsis in low-resource neonatal units using routine healthcare data: Development and evaluation of multivariable statistical and machine learning models
**AUTHORS**	Lowther, Ed; Khan, Nushrat; Cortina-Borja, Mario; Chimhini, Gwendoline; Neal, Samuel; Mangiza, Marcia; Fitzgerald, Felicity; Heys, Michelle; Chimhuya, Simba

VERSION 1 - REVIEW

**REVIEWER NAME**	Lizel Lloyd
**REVIEWER AFFILIATION**	Stellenbosch University Faculty of Medicine and Health Sciences, Department of Paediatrics and Child Health
**REVIEWER CONFLICT OF INTEREST**	N/A
**AI DISCLOSURE**	No
**DATE REVIEW RETURNED**	20-May-2025

**GENERAL COMMENTS**	Dear authors,<br><br>I commend you on publishing this article on a critically important topic: neonatal sepsis.<br><br>A few comments:<br>1. Patient population and burden of infection: The study analysed a large patient cohort, providing a substantial dataset for meaningful statistical evaluation. Of note - based on the gestational age and birth weights of the cohort - there were very few premature infants in this study, a group which carries a higher burden of infection. Future studies should include these vulnerable infants.<br><br>2. Methodological strengths: The statistical methodology was robust, and missing values were thoroughly explained<br><br>3. Contribution to scientific knowledge: A commendable aspect of this study is its inclusion of negative findings, which contribute substantially to broaden the knowledge base.<br><br>4. Study limitations: The study limitations were appropriately addressed, except, in my opinion, the low number of premature infants included in the study. The single time-point for data collection reflects a practical approach, often followed in LRS, but it is sub-optimal. It also needs to be highlighted that this study only investigated EOS.<br><br>5. Future directions: The use of digital tools and machine learning is exciting, with the promise of improved outcomes in future research projects.

**REVIEWER NAME**	Daniel O'Reilly
**REVIEWER AFFILIATION**	Rotunda Hospital, Paediatrics
**REVIEWER CONFLICT OF INTEREST**	N/A
**AI DISCLOSURE**	No
**DATE REVIEW RETURNED**	07-Jul-2025

**GENERAL COMMENTS**	Many thanks for this piece addressing early onset sepsis in a low resource setting, this an area of considerable importance for both proper recognition of neonatal sepsis in areas where blood cultures may take some time and to improve antimicrobial stewardship. Whilst I think fundamentally the principle of this paper is very sound and relevant I have some comments.<br><br>Title: You are only addressing early onset sepsis in this study and this should be made explicit.<br><br>Background: Relevant and well written.<br><br>Methods:<br>1.More detail of what "clinician diagnosed sepsis" looks like is warranted in order to ascertain what that means as even in high income settings this is very variable. Was any sensitivity analysis performed fitting your models using culture proven sepsis as an outcome? PPROM on its own with a single symptom could be "clinician diagnosed sepsis" depending on this classification. If this is not possible it should be acknowledged as an important limitation.<br>2. You discuss hyperparameter tuning for in some detail for lightGBM but not k nearest neighbours beyond "we optimised performance for k". Can you provide greater detail for this? i.e how did you select "reasonable" k<br>3.You have correctly acknowledged the lack of validation dataset and the limitations of your ML which is welcome.<br>4. Table shows categorical variable breakdown as outlined in methods, more information on what signs and symptoms were included i.e. how do danger signs differ from signs of respiratory distress<br>5. Was there any multilevel component to your machine learning analysis e.g did you account for neonatal identity by pregnancy (twins, triplets)? Did you include all multips as independent or account for presumed interdependence? whilst this may be of very little relevance in your cohort (depending on how suspected sepsis is addressed) it should be outlined.<br><br>Overall i think the discussion is written well however I think the "intuitiveness" of knn is perhaps overstated, having attempted to explain this concept previously to a group of clinicians and been left with confused looks. Again how valuable clinician diagnosed sepsis is is also a topic of considerable debate.

VERSION 1 – AUTHOR RESPONSE

Dear authors,

I commend you on publishing this article on a critically important topic: neonatal sepsis.

A few comments:

1. Patient population and burden of infection: The study analysed a large patient cohort, providing a substantial dataset for meaningful statistical evaluation. Of note - based on the gestational age and birth weights of the cohort - there were very few premature infants in this study, a group which carries a higher burden of infection. Future studies should include these vulnerable infants.
Thank you for this comment. As gestational age is often difficult to estimate in this context, we have added details about the numbers of neonates born within the WHO categories for low (n=5063), very low (n=1348) and extremely low birthweight (n=562).

2. Methodological strengths: The statistical methodology was robust, and missing values were thoroughly explained

3. Contribution to scientific knowledge: A commendable aspect of this study is its inclusion of negative findings, which contribute substantially to broaden the knowledge base.
Thank you.

4. Study limitations: The study limitations were appropriately addressed, except, in my opinion, the low number of premature infants included in the study. The single time-point for data collection reflects a practical approach, often followed in LRS, but it is sub-optimal. It also needs to be highlighted that this study only investigated EOS.

Thank you for these comments. We have added some further details on low birthweight, which is a more robust marker than gestational age which is often unreliable in this context. We are in the process of developing daily reviews within Neotree, but this is still in pilot phase at the moment. We have clarified that this work focuses on early onset sepsis within the title.

5. Future directions: The use of digital tools and machine learning is exciting, with the promise of improved outcomes in future research projects.

Thank you, we are also excited!

Reviewer: 2
Dr. Daniel O'Reilly, Rotunda Hospital, University College Dublin
Comments to the Author
Many thanks for this piece addressing early onset sepsis in a low resource setting, this an area of considerable importance for both proper recognition of neonatal sepsis in areas where blood cultures may take some time and to improve antimicrobial stewardship. Whilst I think fundamentally the principle of this paper is very sound and relevant I have some comments.

Title: You are only addressing early onset sepsis in this study and this should be made explicit.

Thank you, we have made this explicit in the title and key messages.

Background: Relevant and well written.

Methods:
1. More detail of what "clinician diagnosed sepsis" looks like is warranted in order to ascertain what that means as even in high income settings this is very variable. Was any sensitivity analysis

performed fitting your models using culture proven sepsis as an outcome? PPROM on its own with a single symptom could be "clinician diagnosed sepsis" depending on this classification. If this is not possible it should be acknowledged as an important limitation.

Thank you for this comment. Unfortunately given the lack of availability of blood cultures, a challenge in many settings similar to ours, clinician-diagnosed sepsis is a pragmatic outcome. We have clarified that by ‘clinician diagnosed sepsis’ we are meaning that on review by either a senior registrar or neonatal consultant after admission, the diagnosis of sepsis was confirmed. This would have been on ward rounds in the days following admission, so the senior clinician will have had the opportunity to observe clinical progress over time, and potentially tests such as full blood counts and (rarely) C-reactive protein to confirm the diagnosis. This is not dissimilar to the process of making a diagnosis of ‘culture-negative’ sepsis in high resource settings, albeit with more laboratory test support. We have also added this to the limitations.

With reference to a sensitivity analysis, absolutely, we did consider modelling with just positive blood culture results as the target. However, given how few culture tests are performed in this setting, our metrics would then be based on finding just ~63 cases in the evaluation dataset; performance would consequently fluctuate according to how the data happened to be divided randomly between training and evaluation sets, and any conclusions drawn may be illusory. Ultimately, for this reason and for the reasons set out above we decided that our composite target variable provides a better benchmark than either of its individual components.

2. You discuss hyperparameter tuning for in some detail for lightGBM but not k nearest neighbours beyond "we optimised performance for k". Can you provide greater detail for this? i.e how did you select "reasonable" k

We have updated the article to make clear that we used the same hyperparameter tuning approach for *k* as for the LightGBM hyperparameters. The search space for *k* was 8 to 512, which is somewhat arbitrary but codifies our belief that relatively large values of *k* should be in scope given the imbalance in our target variable (small values of *k* will often encompass few if any positive cases). In recognition of the validity of your question we have removed the word “reasonable” from this paragraph in the article and have also added a note to clarify that full details of the search spaces for both algorithms are available on the project’s GitHub repository; we would be happy to include full details for ease of reference in an additional supplementary table to the article if this would be useful.

3. You have correctly acknowledged the lack of validation dataset and the limitations of your ML which is welcome.
Thank you.

4. Table shows categorical variable breakdown as outlined in methods, more information on what signs and symptoms were included i.e. how do danger signs differ from signs of respiratory distress

We have clarified in Table 1 that Danger signs are as per WHO Emergency Triage Assessment and Treatment (ETAT) guidelines and include the relevant signs for neonates including grunting, cyanosis, convulsions, capillary refill time < 3 seconds, trunk feels cold, weak femoral pulses.

5. Was there any multilevel component to your machine learning analysis e.g did you account for neonatal identity by pregnancy (twins, triplets)? Did you include all multips as independent or account

for presumed interdependence? whilst this may be of very little relevance in your cohort (depending on how suspected sepsis is addressed) it should be outlined.

Excellent point. Neonatal identity by pregnancy was included in the analysis as a categorical predictor variable, but due to the absence of a maternal ID in the source data we are not currently able to group for multilevel modelling purposes twins or triplets (in cases where more than one were admitted). This is a limitation of the data that we had not previously made clear; we have updated the limitations section accordingly. As you have indicated, the implication of our approach is that in this scenario we treat each multiple as independent. In future research it would be interesting to explore adapting the data schema to enable alternative approaches which include a multilevel component.

Overall I think the discussion is written well however I think the "intuitiveness" of knn is perhaps overstated, having attempted to explain this concept previously to a group of clinicians and been left with confused looks. Again how valuable clinician diagnosed sepsis is also a topic of considerable debate.

Thank you for this. Neonatal sepsis research globally, not just in low-resource settings, has been hampered by the limitations of blood cultures as a gold standard (https://www.thelancet.com/journals/lancet/article/PIIS0140-6736(24)00495-1/). This is compounded by the challenges specific to performing blood cultures in low-resource settings, with challenges at every step from taking the test, to performing the test, interpreting and feeding back the results. While we and others are taking incremental steps towards improving this situation (https://journals.plos.org/globalpublichealth/article?id=10.1371/journal.pgph.0004343) there is still a long way to go, so we have chosen to pragmatically use senior clinician diagnosis as an outcome within this phase of modelling. As we state, this paper is proof of concept, and further investigation – including testing our assumption about the intuitiveness of knn among clinicians – will be vital before our approach is ready for clinical use.

VERSION 2 - REVIEW

**REVIEWER NAME**	Daniel O'Reilly
**REVIEWER AFFILIATION**	Rotunda Hospital, Paediatrics
**REVIEWER CONFLICT OF INTEREST**	N/A
**AI DISCLOSURE**	No
**DATE REVIEW RETURNED**	22-Aug-2025

**GENERAL COMMENTS**	Many thanks for addressing my previous comments, the clarity around modelling and definition is helpful and I hope will help allow other authors working on this important issue to put your work in context.
